# Supplementary material for: Single-cell morphometrics reveals T-box gene-dependent patterns of epithelial tension in the Second Heart field
Source: Nat Commun. 2024 Nov 4;15:9512. doi: 10.1038/s41467-024-53612-8 (PMC11535409; doi:10.1038/s41467-024-53612-8)
Supplement: Supplementary file 2 — Reporting Summary [file 41467_2024_53612_MOESM2_ESM.pdf]

nature portfolio

Corresponding author(s): Robert G. Kelly and Paul Villoutreix

Last updated by author(s): 26 September 2024

Reporting Summary

Nature Portfolio wishes to improve the reproducibility of the work that we publish. This form provides structure for consistency and transparency in reporting. For further information on Nature Portfolio policies, see our Editorial Policies and the Editorial Policy Checklist.

Statistics

For all statistical analyses, confirm that the following items are present in the figure legend, table legend, main text, or Methods section.

n/a	Confirmed	
☐	☒	The exact sample size (*n*) for each experimental group/condition, given as a discrete number and unit of measurement
☐	☒	A statement on whether measurements were taken from distinct samples or whether the same sample was measured repeatedly
☐	☒	The statistical test(s) used AND whether they are one- or two-sided<br>*Only common tests should be described solely by name; describe more complex techniques in the Methods section.*
☐	☒	A description of all covariates tested
☐	☒	A description of any assumptions or corrections, such as tests of normality and adjustment for multiple comparisons
☐	☒	A full description of the statistical parameters including central tendency (e.g. means) or other basic estimates (e.g. regression coefficient) AND variation (e.g. standard deviation) or associated estimates of uncertainty (e.g. confidence intervals)
☐	☒	For null hypothesis testing, the test statistic (e.g. *F*, *t*, *r*) with confidence intervals, effect sizes, degrees of freedom and *P* value noted<br>*Give P values as exact values whenever suitable.*
☒	☐	For Bayesian analysis, information on the choice of priors and Markov chain Monte Carlo settings
☒	☐	For hierarchical and complex designs, identification of the appropriate level for tests and full reporting of outcomes
☒	☐	Estimates of effect sizes (e.g. Cohen's *d*, Pearson's *r*), indicating how they were calculated

Our web collection on statistics for biologists contains articles on many of the points above.

Software and code

Policy information about availability of computer code

Data collection	Imaging data was acquired under Zeiss LSM780 or 880 confocal microscopes for wholemount immunofluorescences.<br>Image processing was performed Image J 1.53c and Imaris 10.1.1 softwares.<br>Image analysis and quantification was performed using Tissue Analyzer v23 plug-in for FIJI, LocalZ projector v1.5.4 plug-in for FIJI, Deproj plug-in forMATLAB, Force inference 2019-10 plug in for MATLAB and Python 3.7.9.<br>Panels figures and schemes were made using Adobe Illustrator 24.0.2.
Data analysis	The measurement of the Golgi-to-nucleus polarity in 3D and the sc-morphology analysis is available on: https://github.com/VILLOUTREIXLab/sc-morphometrics-SHF, 2) the code of the Bayesian force-inference has been published (Kong, 2019), 3) the Joint Spatial PCA is available on the Github: https://github.com/VILLOUTREIXLab/JointSpatialPCA

For manuscripts utilizing custom algorithms or software that are central to the research but not yet described in published literature, software must be made available to editors and reviewers. We strongly encourage code deposition in a community repository (e.g. GitHub). See the Nature Portfolio guidelines for submitting code & software for further information.

Data

Policy information about availability of data

All manuscripts must include a data availability statement. This statement should provide the following information, where applicable:

- Accession codes, unique identifiers, or web links for publicly available datasets
- A description of any restrictions on data availability
- For clinical datasets or third party data, please ensure that the statement adheres to our policy

Source data are provided with this paper as a Source Data file and available on the Github: https://github.com/VILLOUTREIXLab/sc-morphometrics-SHF.

Research involving human participants, their data, or biological material

Policy information about studies with human participants or human data. See also policy information about sex, gender (identity/presentation), and sexual orientation and race, ethnicity and racism.

Reporting on sex and gender	N/A
Reporting on race, ethnicity, or other socially relevant groupings	N/A
Population characteristics	N/A
Recruitment	N/A
Ethics oversight	N/A

Note that full information on the approval of the study protocol must also be provided in the manuscript.

Field-specific reporting

Please select the one below that is the best fit for your research. If you are not sure, read the appropriate sections before making your selection.

☒ Life sciences ☐ Behavioural & social sciences ☐ Ecological, evolutionary & environmental sciences

For a reference copy of the document with all sections, see nature.com/documents/nr-reporting-summary-flat.pdf

Life sciences study design

All studies must disclose on these points even when the disclosure is negative.

Sample size	No statistical method was used to determine the sample size. Sample sizes were selected based on the experiment type and the standard practices in the field of genetics and stem cell biology.
Data exclusions	No data exclusions
Replication	All experiments have been repeated in at least three biological replicates per genotype or stage. The exact numbers are clearly indicated in the figures legends. All experimental findings were reliably reproduced.
Randomization	Samples were acquired by developmental stage and genotype. The data was then analyzed in an automatized manner.<br>The samples were allocated depending on the embryos stage, number of somites and headfold development as described in LeGarrec et al.2017.
Blinding	The investigator was not blinded during embryo collection but all measurements were quantified using unsupervised machine learning.

Reporting for specific materials, systems and methods

We require information from authors about some types of materials, experimental systems and methods used in many studies. Here, indicate whether each material, system or method listed is relevant to your study. If you are not sure if a list item applies to your research, read the appropriate section before selecting a response.

Materials & experimental systems

n/a	Involved in the study
☐	☒ Antibodies
☒	☐ Eukaryotic cell lines
☒	☐ Palaeontology and archaeology
☐	☒ Animals and other organisms
☒	☐ Clinical data
☒	☐ Dual use research of concern
☒	☐ Plants

Methods

n/a	Involved in the study
☒	☐ ChIP-seq
☒	☐ Flow cytometry
☒	☐ MRI-based neuroimaging

Antibodies

Antibodies used

The following antibodies were used: goat anti-Tbx5 (1/250, Santa Cruz sc-7866), rabbit anti-Cre (1/500, Novagen 69050), rabbit anti-ppMLC (1/200, Cell Signaling Thr18/Ser19 3674), mouse anti-Golgi (GM-130) (1/100, BD Transduction Laboratories 610823). Fluorescent secondary antibodies Alexa 488, 568 and 647 from Jackson and Invitrogen were used at 1/200. Two fluorescent forms of Phalloidin were used to label F-actin: phalloidin 647 (1/50, Sigma 65906) or phalloidin 488 (1/50, Sigma 49409).

Validation

All commercial antibodies were validated by the manufacturer for the species and application used in the study. Some of the antibodies have already been used in previous studies Francou et al. 2017, de Bono et al. 2018.

Animals and other research organisms

Policy information about studies involving animals; ARRIVE guidelines recommended for reporting animal research, and Sex and Gender in Research

Laboratory animals

The strains used in this study were previously described in:
Wild-type CD1 mice: E8.5 N =11; E9.5 N=20
Tbx1-/-: Jerome et al.2011 DiGeorge syndrome phenotype in mice mutant for the T-box gene, Tbx1. Nat. Genet. 27, 286–291 (2001). E9.5 N=4
Tbx5fl/fl: Bruneau et al.2001. A murine model of Holt-Oram syndrome defines roles of the T-box transcription factor Tbx5 in cardiogenesis and disease. Cell 106, 709–721 (2001). E9.5 N =9
Mef2c-AHF-Cre: The right ventricle, outflow tract, and ventricular septum comprise a restricted expression domain within the secondary/anterior heart field. Dev. Biol. 287, 134–145 (2005). E9.5 N =9
Nkx2.5-/- mice were described in previous studies from Tanaka, M. et al. 1999. The cardiac homeobox gene Csx/Nkx2.5 lies genetically upstream of multiple genes essential for heart development. Development 126, 1269–1280 (1999). N=4

The mice have been added in the manuscript and the primers in the Supplementary Table1.

Wild animals

The study did not involve wild animals.

Reporting on sex

Our data have not considered sex or gender that is not reported to impact on the phenotypes evaluated in this study.

Field-collected samples

N/A

Ethics oversight

All studies and procedures involving animals were in strict accordance with the recommendations of the European Community Directive (2010/63/UE) for the protection of vertebrate animals used for experimental and other scientific purposes. The project was specifically approved by the ethics committee of the IBDM SBEA and by the French Ministry of Research (Apafis No. 10266-2017061618121519). Husbandry, supply of animals, as well as maintenance and care of the animals in the Animal Facility of CNRS-IBDM (facility license #G-13 055 21) before and during experiments fully satisfied the animals' needs and welfare.

Note that full information on the approval of the study protocol must also be provided in the manuscript.

Plants

Seed stocks	N/A
Novel plant genotypes	N/A
Authentication	N/A
