# Supplementary material for: Single-cell morphometrics reveals T-box gene-dependent patterns of epithelial tension in the Second Heart field
Source: Nat Commun. 2024 Nov 4;15:9512. doi: 10.1038/s41467-024-53612-8 (PMC11535409; doi:10.1038/s41467-024-53612-8)
Supplement: Supplementary file 1 — Supplementary Information [file 41467_2024_53612_MOESM1_ESM.pdf]

Supplementary Information

Single-cell morphometrics reveals T-box dependent patterns of epithelial tension in the Second Heart field

Clara Guijarro[1,2,3 +], Solène Song[2,3 +], Benoit Aigouy[1], Raphael Clement[1], Paul Villoutreix[2,3 *], Robert G. Kelly[1 *]

[1] Aix-Marseille Université, CNRS UMR 7288, IBDM, Turing Centre for Living systems, Marseille, France

[2] Aix-Marseille Université, LIS, UMR 7020, Turing Centre for Living systems, Marseille, France

[3] Aix-Marseille Université, MMG, Inserm U1251, Turing Centre for Living systems, Marseille, France

[+] These authors contributed equally

[*] These authors jointly supervised this work

Addresses for correspondence:
Robert G. Kelly,
Aix-Marseille University, CNRS UMR 7288,
IBDM, Luminy Campus Case 907,
73288 Marseille Cedex 9, France
E-mail: Robert.Kelly@univ-amu.fr

Paul Villoutreix,
Marseille Medical Genetics Inserm U 1251,
Turing Centre for Living Systems,
Aix-Marseille University, La Timone Campus,
13005 Marseille, France
E-mail: Paul.Villoutreix@univ-amu.fr

Figure S1

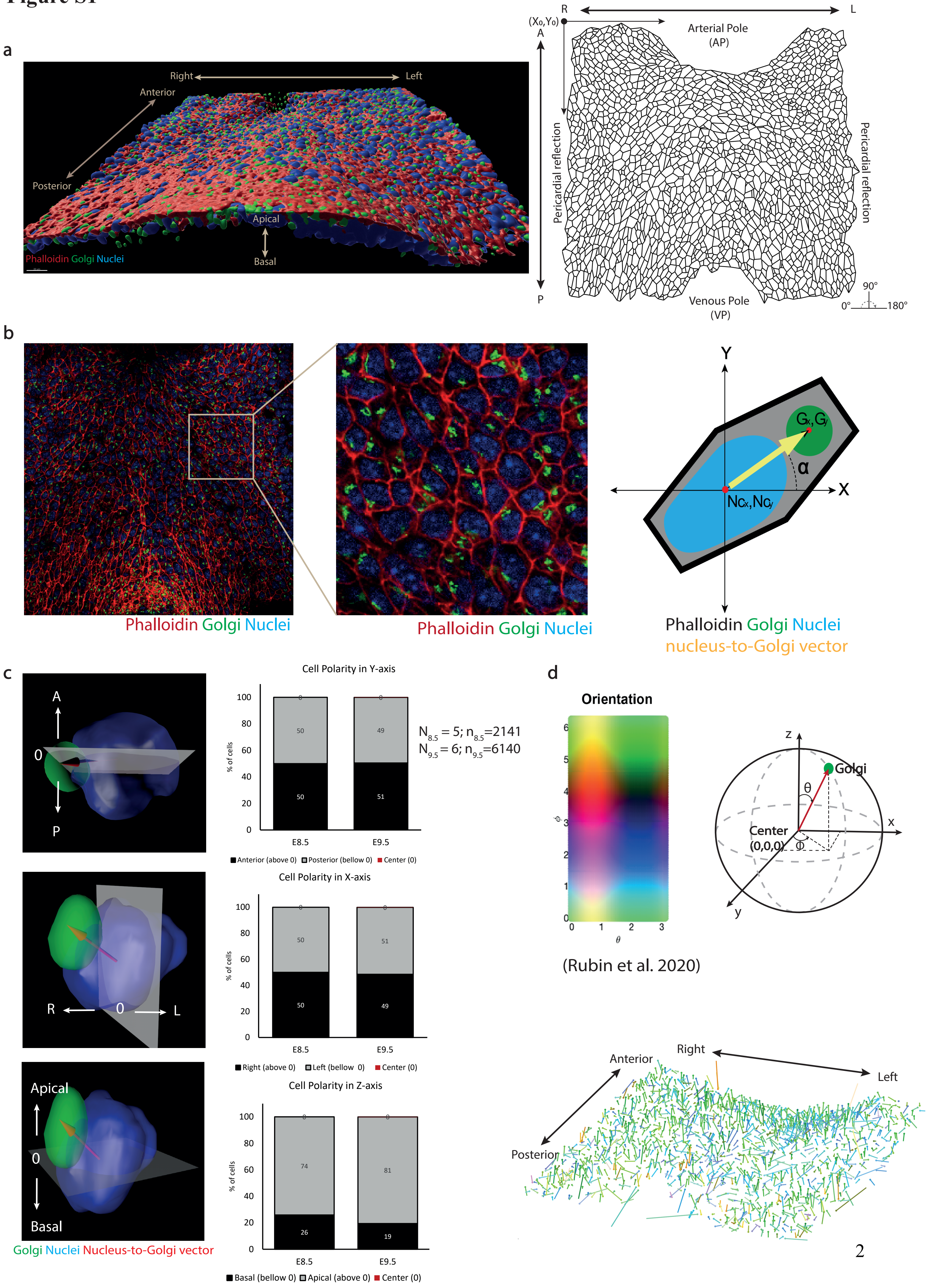
a
Right
Left
Anterior
Posterior
Apical
Basal
Phalloidin Golgi Nuclei
R
L
(X0,Y0)
A
P
Arterial Pole
(AP)
Pericardial reflection
Pericardial reflection
Venous Pole
(VP)
90°
0°
180°
b
Phalloidin Golgi Nuclei
Phalloidin Golgi Nuclei
Y
X
Gx,Gy
α
Ncx,Ncy
Phalloidin Golgi Nuclei
nucleus-to-Golgi vector
c
A
0
P
R
0
L
Apical
0
Basal
Golgi Nuclei Nucleus-to-Golgi vector
Cell Polarity in Y-axis
% of cells
E8.5
E9.5
Anterior (above 0)
Posterior (bellow 0)
Center (0)
N8.5 = 5; n8.5=2141
N9.5 = 6; n9.5=6140
Cell Polarity in X-axis
Right (above 0)
Left (bellow 0)
Center (0)
Cell Polarity in Z-axis
Basal (bellow 0)
Apical (above 0)
Center (0)
d
Orientation
φ
θ
z
Golgi
θ
x
Center
(0,0,0)
Φ
y
(Rubin et al. 2020)
Anterior
Right
Left
Posterior

Fig. S1: Characterization of the nucleus-to-Golgi polarity in cells of the dorsal pericardial wall. a. The E9.5 dorsal pericardial wall visualized after 3D (left) and apical (right) segmentation, showing the axes and coordinates referred to in this study. Phalloidin (red), Golgi (green), nuclei (blue). **b.** Maximum intensity projection of the apical part of the dorsal pericardial wall epithelium, high magnification of the epithelium corresponding to the boxed region and scheme of the calculation of the nucleus-to Golgi polarity used for single-cell morphometrics (the angle formed between the vector from the center of the nucleus to the center of the Golgi and the left-right position (X) within the tissue. **c.** Stack count-plots of the localization of nucleus-to Golgi polarity in 3D. Golgi (green), nuclei (blue), nucleus-to-golgi vector (red) **d.** Spatial distribution of the vectors of each cell of the epithelium color coded according to the angle as already described in Rubin et al., 2020[30]. A: anterior; P: posterior; R: right; L: left. Source data are provided as a Source Data file. E8.5 N=5 embryos, E8.5 n=2141cells ; E9.5 N=4 embryos, E9.5 n= 6140 cells.

Figure S2

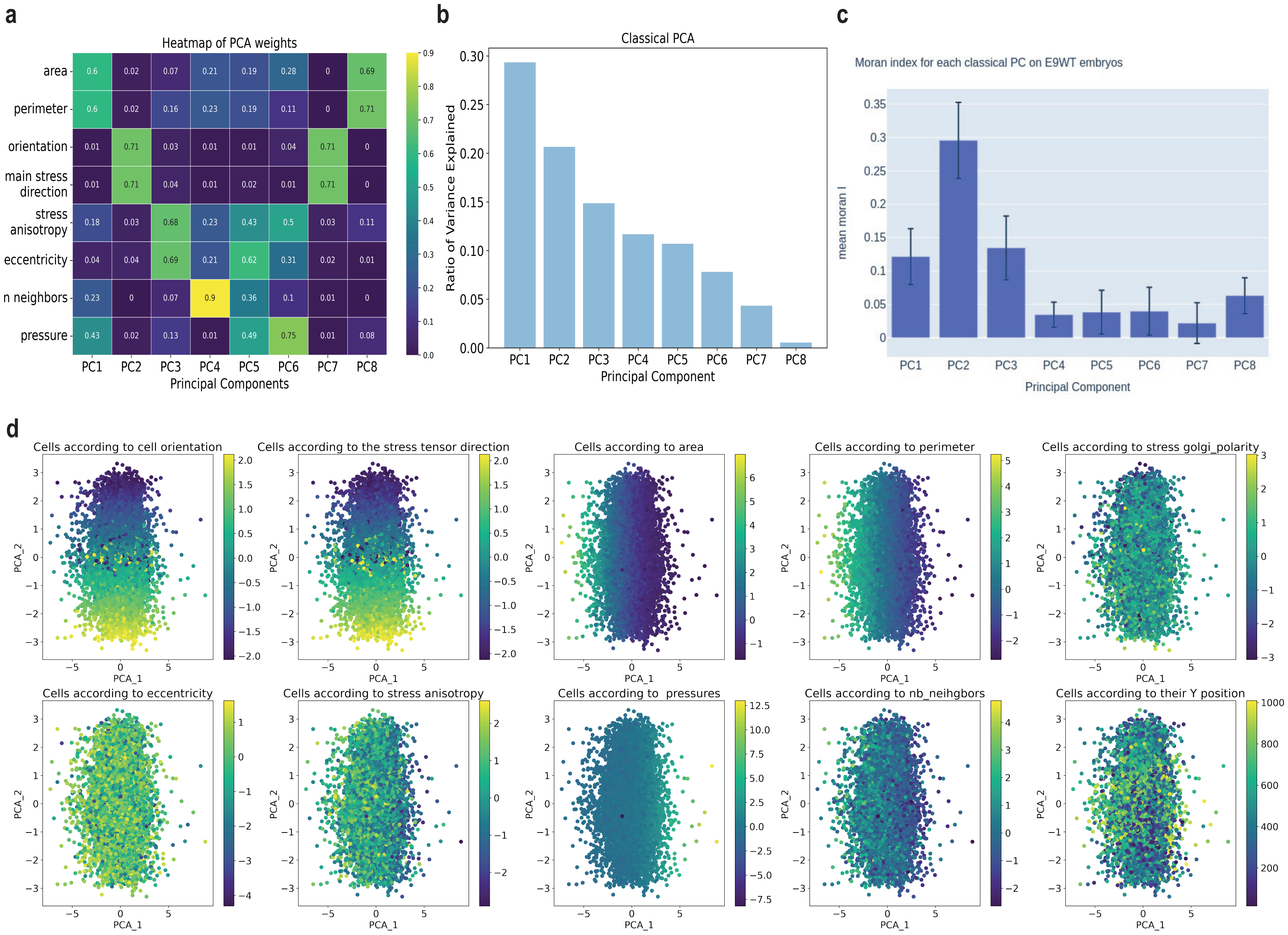

Fig. S2: Principal component analysis of cells in the dorsal pericardial wall of E9.5 wild-type embryos. a. Heatmap showing the coefficients of each feature for each principal component (PCA). **b.** Bar plot showing the PCA explained variance. **c**. Moran index for each classical PC on E9.5 wild-type embryos. **d.** PCA visualization using PC1 as x-axis and PC2 as y-axis and color coded depending on the dispersion of the values for each feature. Source data are provided as a Source Data file. Error bars represent SD. N = 6 embryos, n = 8618 cells.

Figure S3

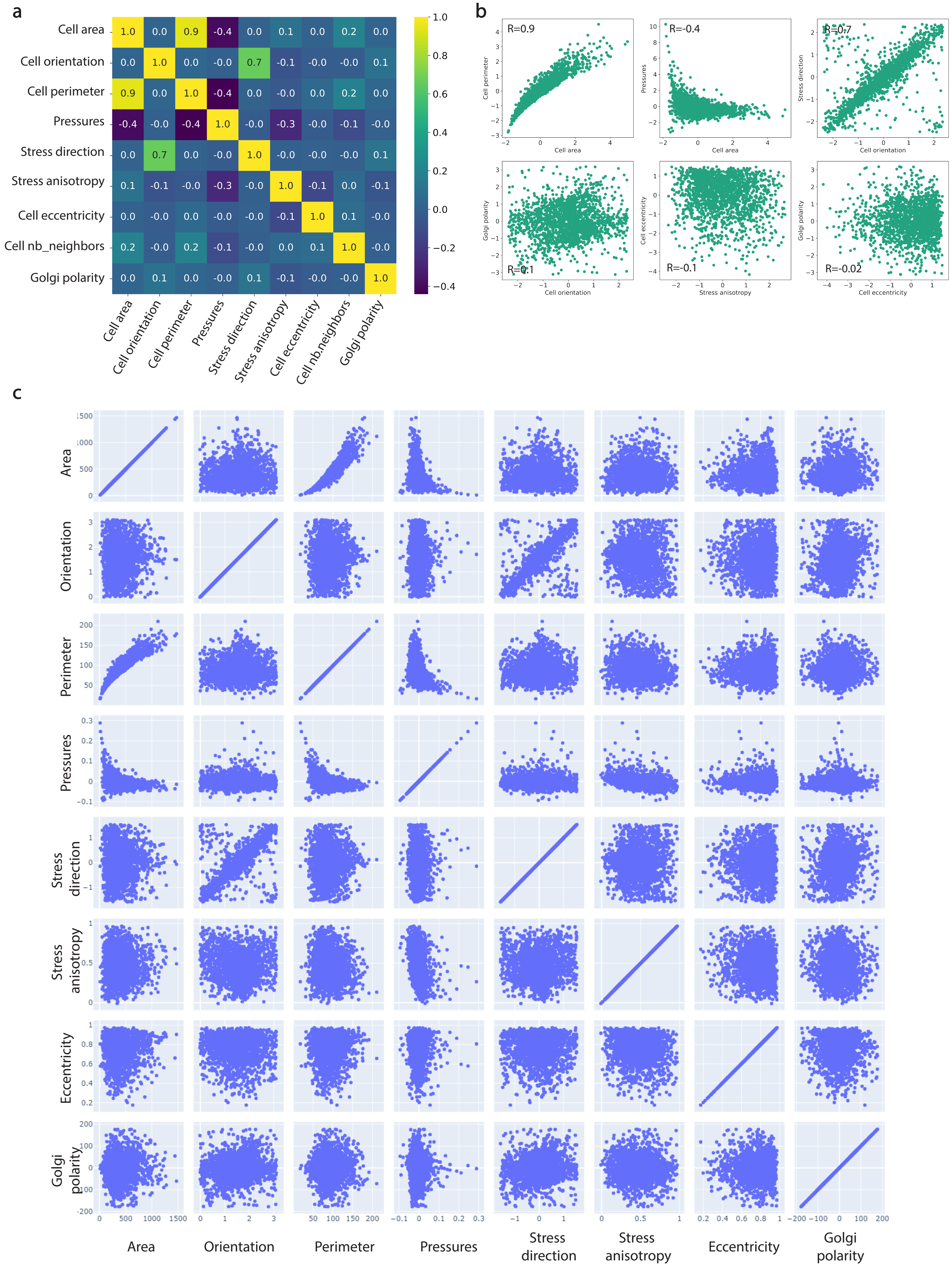
a
Cell area
Cell orientation
Cell perimeter
Pressures
Stress direction
Stress anisotropy
Cell eccentricity
Cell nb_neighbors
Golgi polarity
1.0 0.0 0.9 -0.4 0.0 0.1 0.0 0.2 0.0
0.0 1.0 0.0 -0.0 0.7 -0.1 -0.0 -0.0 0.1
0.9 0.0 1.0 -0.4 0.0 -0.0 0.0 0.2 0.0
-0.4 -0.0 -0.4 1.0 -0.0 -0.3 -0.0 -0.1 -0.0
0.0 0.7 0.0 -0.0 1.0 -0.0 -0.0 -0.0 0.1
0.1 -0.1 -0.0 -0.3 -0.0 1.0 -0.1 0.0 -0.1
0.0 -0.0 0.0 -0.0 -0.0 -0.1 1.0 0.1 -0.0
0.2 -0.0 0.2 -0.1 -0.0 0.0 0.1 1.0 -0.0
0.0 0.1 0.0 -0.0 0.1 -0.1 -0.0 -0.0 1.0
Cell area
Cell orientation
Cell perimeter
Pressures
Stress direction
Stress anisotropy
Cell eccentricity
Cell nb.neighbors
Golgi polarity
b
R=0.9
R=-0.4
R=0.7
R=0.1
R=-0.1
R=-0.02
Cell perimeter
Cell area
Pressures
Stress direction
Cell orientation
Golgi polarity
Cell eccentricity
Stress anisotropy
c
Area
Orientation
Perimeter
Pressures
Stress direction
Stress anisotropy
Eccentricity
Golgi polarity

Fig. S3: Feature correlations. a. Correlation matrix. **b.** Correlation plots per features (only the most correlated are shown). **c.** Correlation graphs between all features. Source data are provided as a Source Data file.

Figure S4

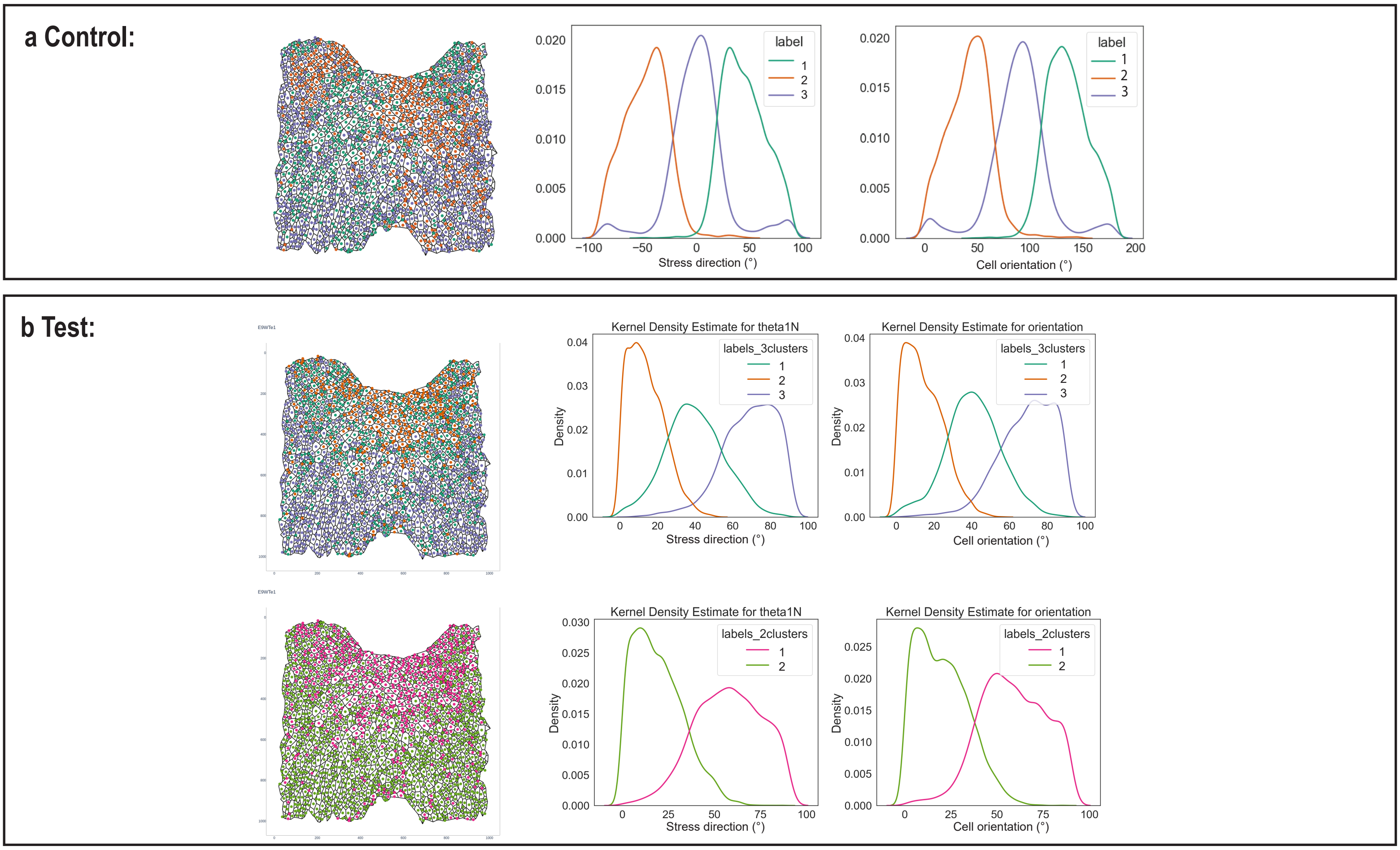

c

n	AIC
1	28532
2	28380
3	28366
4	28389
5	28377
6	28374
7	28382
8	28376
9	28377

Fig. S4: Cluster signatures in E9.5 wild-type. a. Cluster maps and kernel density distribution plots for stress direction and orientation using the all the raw values (control). **b.** Cluster maps and kernel density distribution plots using the AP-symmetrized values of orientation and stress direction for three and two clusters. **c.** Optimal number of clusters according to Akaike Information Criterion (AIC). N = 6 embryos, n = 8618 cells. Source data are provided as a Source Data file.

Figure S5

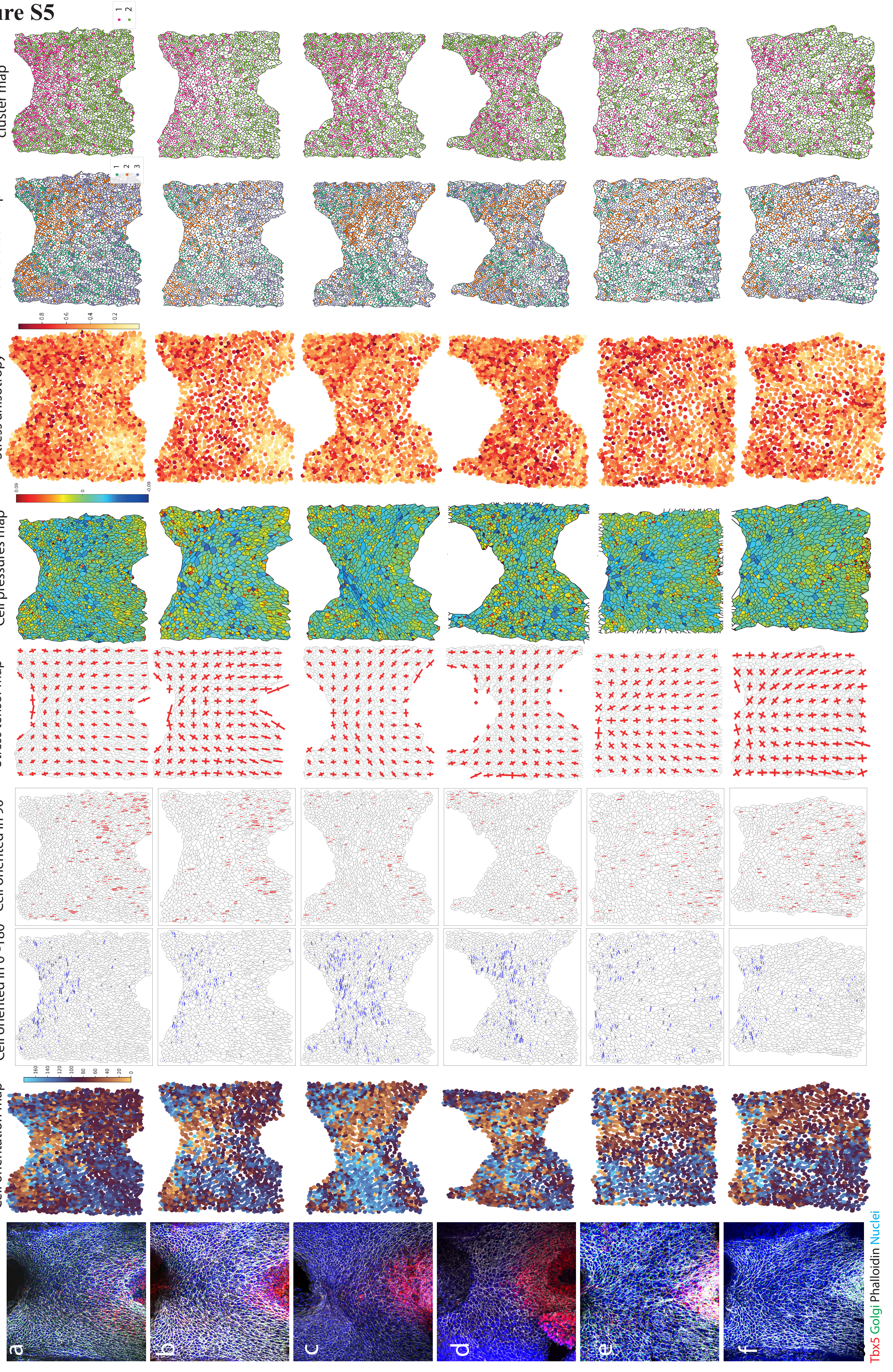

Symmetrized cluster map
Cell cluster map
Stress anisotropy
Cell pressures map
Stress tensor map
Cell oriented in 90°
Cell oriented in 0°-180°
Cell orientation map
WM- IF
a
b
c
d
e
f
Tbx5 Golgi Phalloidin Nuclei

Fig. S5: Morphometrics reproducibility in E9.5 wild-type embryos. a-f. Showing, for each embryo, in the following order: the apical projection of the dorsal pericardial wall after whole-mount immunofluorescence (Tbx5 in red, phalloidin in white, nuclei in blue), the cell orientation map, cells oriented horizontally (in blue) along the left-right axis of the embryo on the segmentation channel, cells oriented vertically (in red) along the anterior-posterior axis of the embryo, the stress tensor map, the cell pressure map, stress anisotropy map and cell cluster distribution shown for three clusters and two clusters after symmetrizing for cell orientation and stress direction. Embryos a-d have >20 somites and embryos e and f < 20 somites. Embryo f has not been stained with TBX5 antibody. N = 6 embryos, n = 8618 cells.

Figure S6

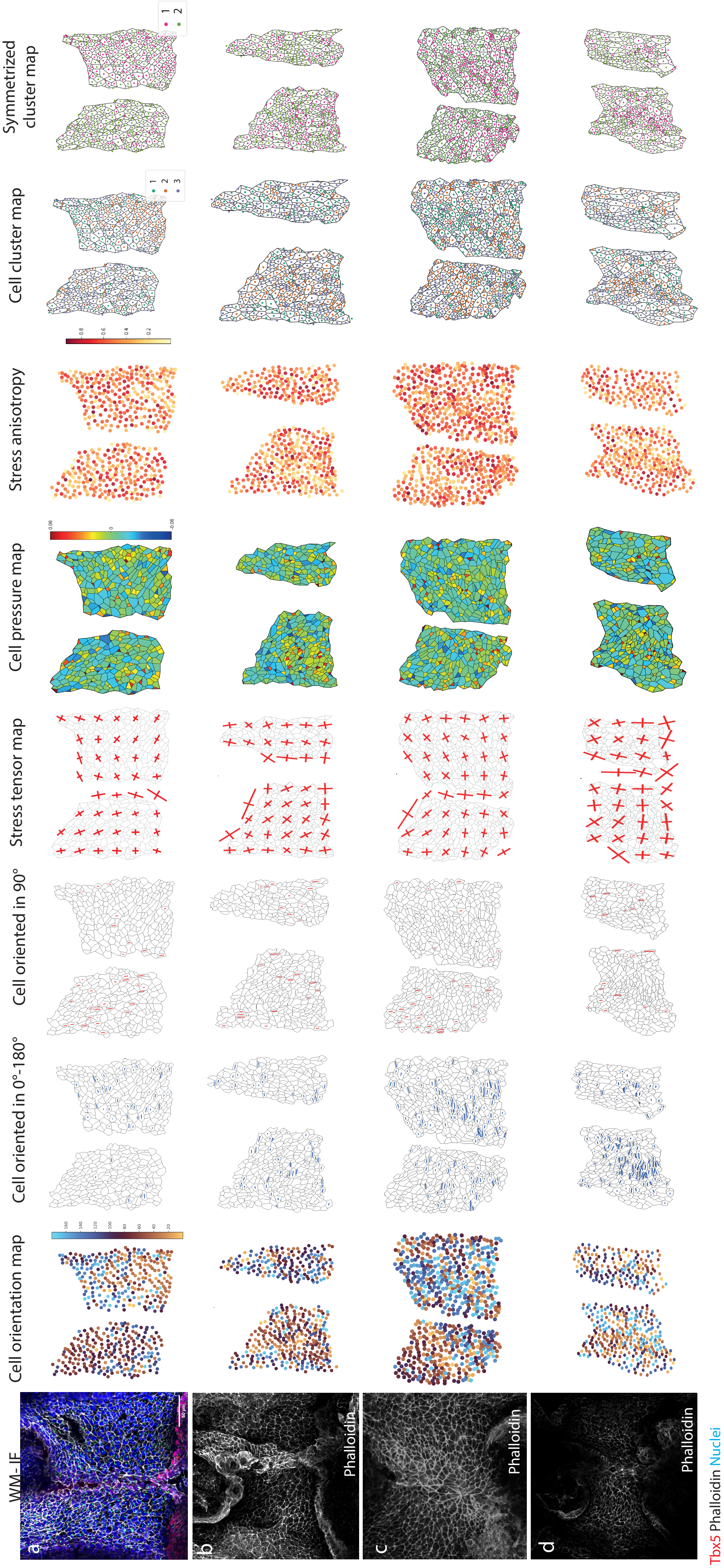

Symmetrized cluster map
Cell cluster map
Stress anisotropy
Cell pressure map
Stress tensor map
Cell oriented in 90°
Cell oriented in 0°-180°
Cell orientation map
WM-IF
a
b
c
d
Phalloidin
Tbx5 Phalloidin Nuclei

Fig. S6: Morphometrics in E8.5 wild-type embryos. a-d. Showing for each embryo, in the following order, the apical projection of the dorsal pericardial wall after whole-mount immunofluorescence (TBX5 in red, phalloidin in white, nuclei in blue), the cell orientation map, cells oriented horizontally (in blue) along the left-right axis of the embryo on the segmentation channel, cells oriented vertically (in red) along the anterior-posterior axis of the embryo, the stress tensor map, the cell pressure map, stress anisotropy map and cell cluster distribution shown for three clusters and two clusters after symmetrizing for cell orientation and stress direction. N = 6 embryos, n = 2994 cells.

Figure S7

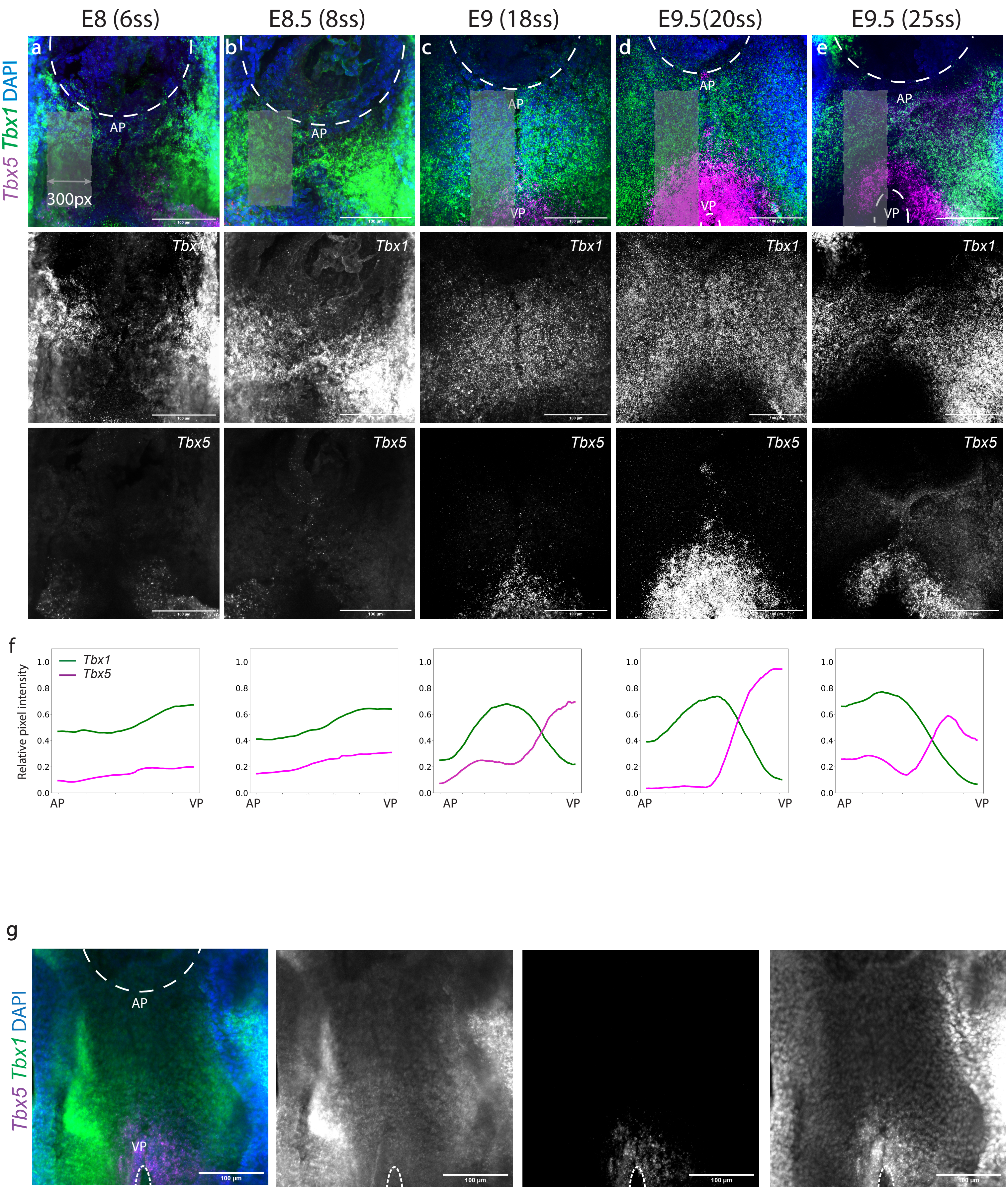
E8 (6ss)
E8.5 (8ss)
E9 (18ss)
E9.5(20ss)
E9.5 (25ss)
Tbx5 Tbx1 DAPI
a
b
c
d
e
AP
VP
300px
Tbx1
Tbx5
f
Relative pixel intensity
Tbx1
Tbx5
1.0
0.8
0.6
0.4
0.2
0.0
AP
VP
g
Tbx5 Tbx1 DAPI
100 µm

Fig. S7: *Tbx1* and *Tbx5* mRNA distribution and quantification from E8 to E9.5. a-e. Frontal whole-mount views of the dorsal pericardial wall of wildtype embryos after RNAscope fluorescent in situ hybridization showing nuclei (blue), *Tbx1* (green) and *Tbx5* (magenta) mRNA distribution between E8 (6 somites) and E9.5 (25 somites) wild-type embryos, before and after dorsal mesocardial breakdown. The white line delimits the arterial pole of the heart (AP). Grey areas represent the area where the pixel intensity has been measured. **f.** Quantification of mRNA pixel intensity of *Tbx1* and *Tbx5*. **g.** Frontal whole-mount views of the dorsal pericardial wall of *Mef2cAHFCre;Tbx5*$^{fl/fl}$ embryos after RNAscope fluorescent in situ hybridization showing nuclei (blue), *Tbx1* (green) and *Tbx5* (magenta) mRNA distribution. Quantification areas have a width of 300 pixels. Scale bars:100µm. Source data are provided as a Source Data file. E8-E8.5 : N = 5 embryos, E9 : N = 3 embryos, E9.5 : N = 6 embryos.

Figure S8

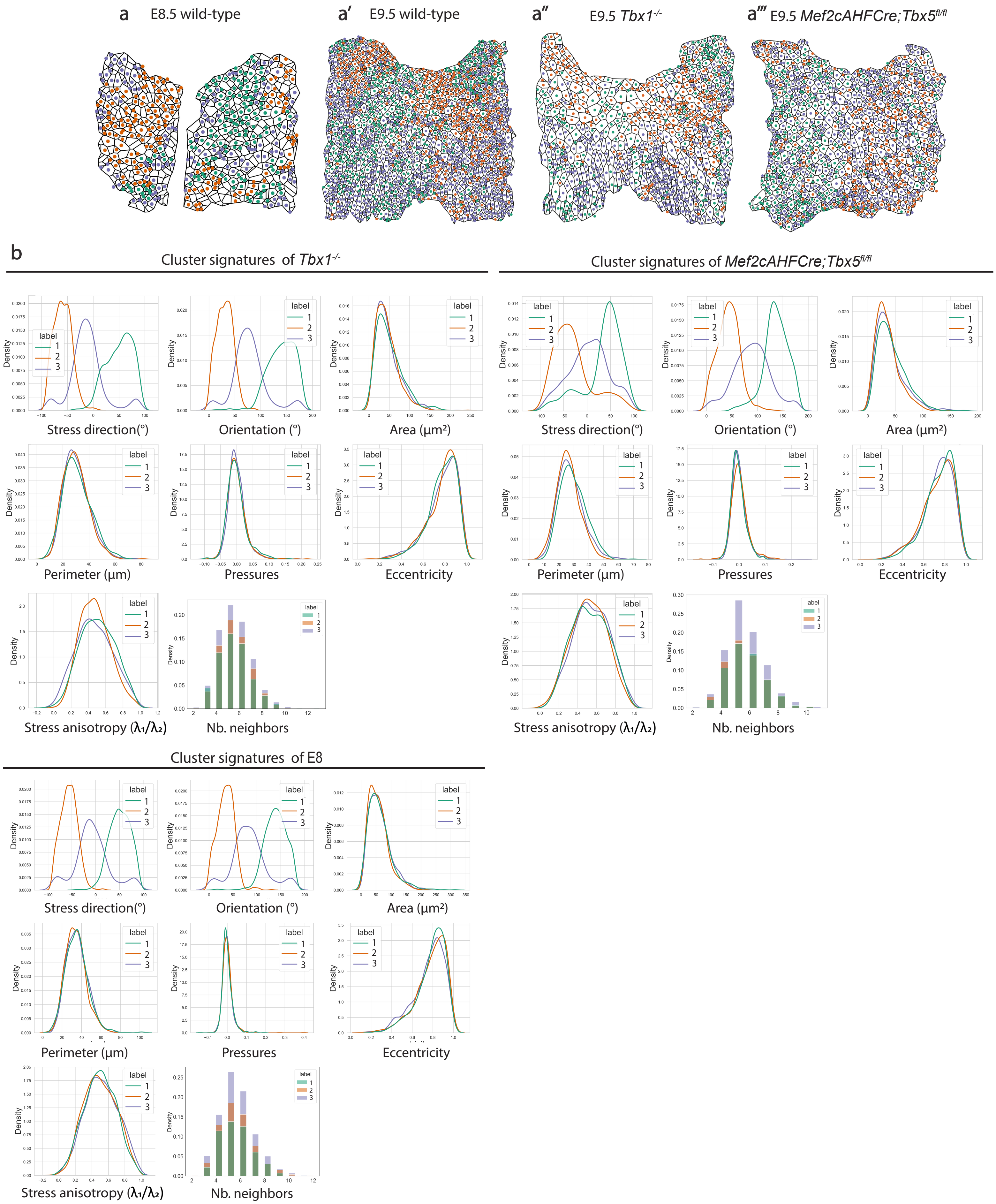

a E8.5 wild-type
a′ E9.5 wild-type
a″ E9.5 Tbx1-/-
a‴ E9.5 Mef2cAHFCre;Tbx5fl/fl
b
Cluster signatures of Tbx1-/-
Cluster signatures of Mef2cAHFCre;Tbx5fl/fl
Cluster signatures of E8
Stress direction(°)
Orientation (°)
Area (μm²)
Perimeter (μm)
Pressures
Eccentricity
Stress anisotropy (λ1/λ2)
Nb. neighbors
Density
label
1
2
3

Fig. S8: Morphometrics of E9.5, 8.5 wildtype and E9.5 *Tbx1* and *Tbx5* conditional mutant embryos. a-a"'. Cluster distribution in real space in wild-type E8.5 and E9.5 embryos, E9.5 *Tbx1*$^{-/-}$ and *Mef2cAHFCre;Tbx5*$^{fl/fl}$ embryos. **b.** Cluster signatures and kernel density distributions of *Tbx1*$^{-/-}$, *Mef2cCreAHFCre*; *Tbx5*$^{fl/fl}$ and E8.5 wild-type embryos. Source data are provided as a Source Data file. E8.5 wild-type: N = 6 embryos, n = 2994 cells; E9.5 wild-type: N = 6 embryos, n = 8618 cells; E9.5 *Tbx1*$^{-/-}$: N = 3 embryos, n = 2551; E9.5 *Mef2cAHFCre;Tbx5*$^{fl/fl}$: N = 4 embryos, n = 2809 cells.

Figure S9

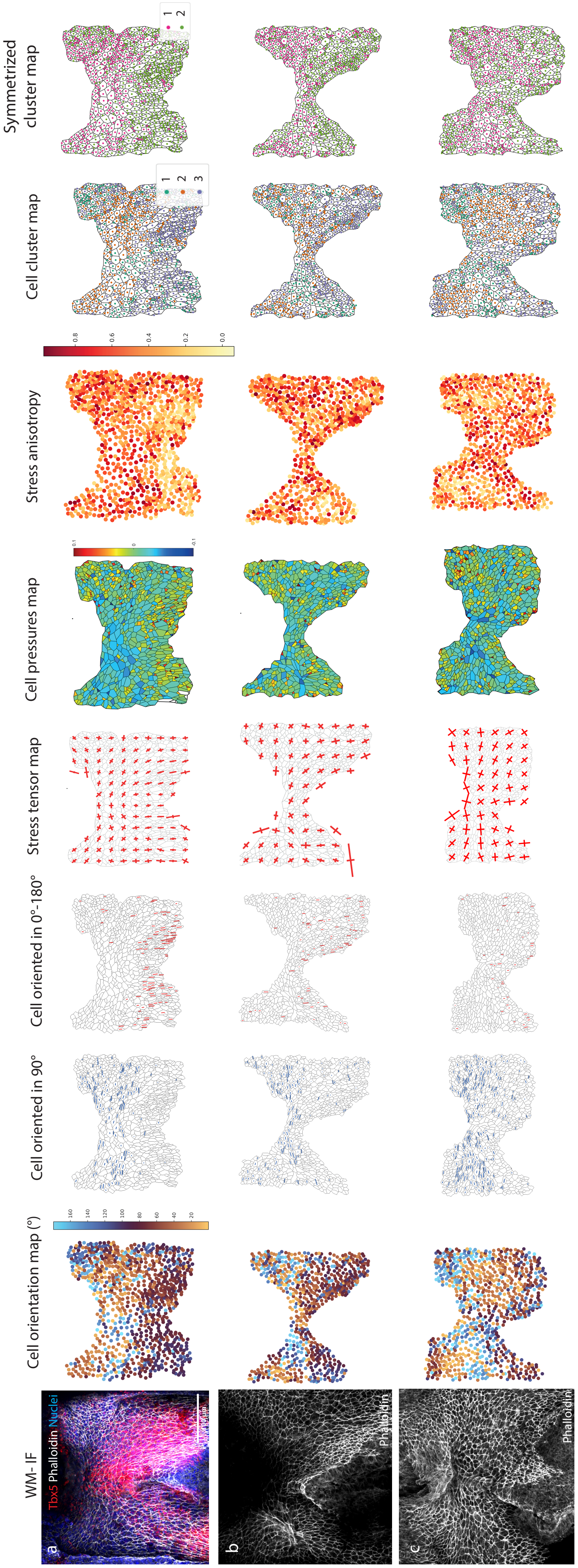

WM- IF
Cell orientation map (°)
Cell oriented in 90°
Cell oriented in 0°-180°
Stress tensor map
Cell pressures map
Stress anisotropy
Cell cluster map
Symmetrized cluster map
a
Tbx5 Phalloidin Nuclei
b
Phalloidin
c
Phalloidin

Fig. S9: Morphometrics reproducibility in E9.5 *Tbx1$^{-/-}$* embryos. a-c. Showing, for each embryo, in the following order: the apical projection of the dorsal pericardial wall after whole-mount immunofluorescence (TBX5 in red, phalloidin in white, nuclei in blue), the cell orientation map, cells oriented horizontally (in blue) along the left-right axis of the embryo on the segmentation channel, cells oriented vertically (in red) along the anterior-posterior axis of the embryo, the stress tensor map, the cell pressure map, the stress anisotropy map and cell cluster distribution shown for three clusters and two clusters after symmetrizing for cell orientation and stress direction. N = 3 embryos, n = 2551 cells.

Figure S10

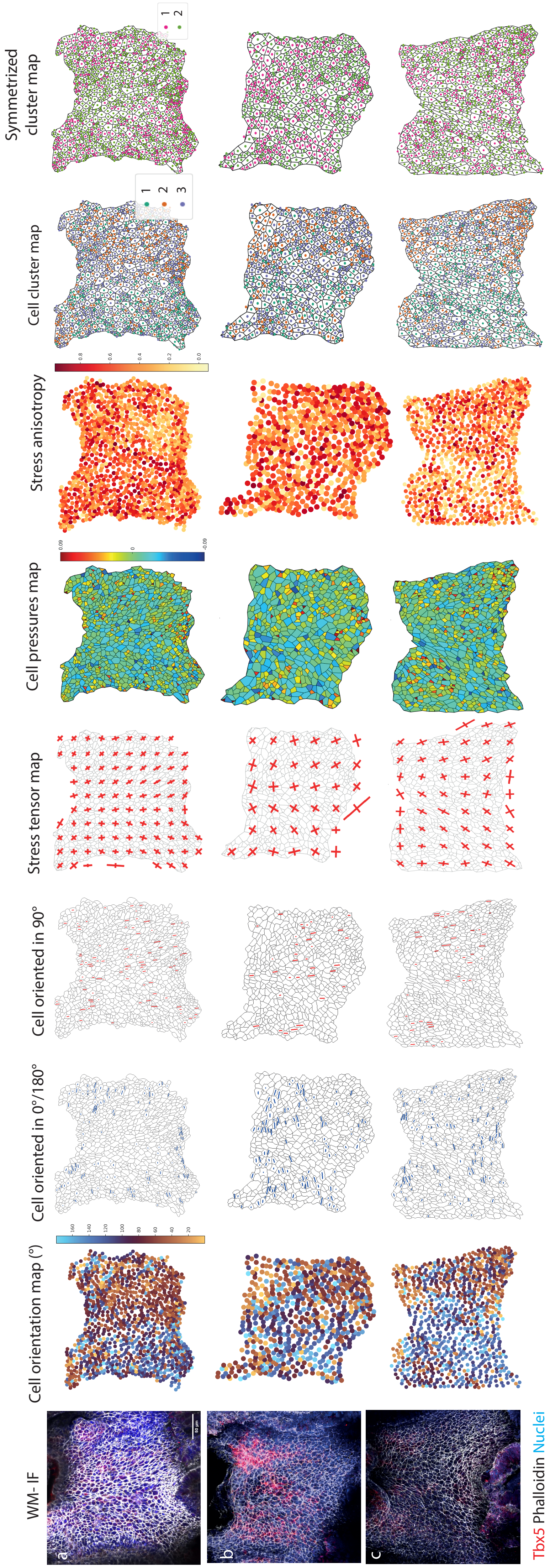

WM- IF
Cell orientation map (°)
Cell oriented in 0°/180°
Cell oriented in 90°
Stress tensor map
Cell pressures map
Stress anisotropy
Cell cluster map
Symmetrized cluster map
a
b
c
Tbx5 Phalloidin Nuclei

Fig. S10: Morphometrics reproducibility in E9.5 *Mef2cAHFCre;Tbx5$^{fl/fl}$* embryos. a-c. Showing, for each embryo, in the following order: the apical projection of the dorsal pericardial wall after whole-mount immunofluorescence (TBX5 in red, phalloidin in white, nuclei in blue), the cell orientation map, cells oriented horizontally (in blue) along the left-right axis of the embryo on the segmentation channel, cells oriented vertically (in red) along the anterior-posterior axis of the embryo, the stress tensor map, the cell pressure map, the stress anisotropy map and cell cluster distribution shown for three clusters and two clusters after symmetrizing for cell orientation and stress direction. N = 4 embryos, n = 2809 cells.

Figure S11

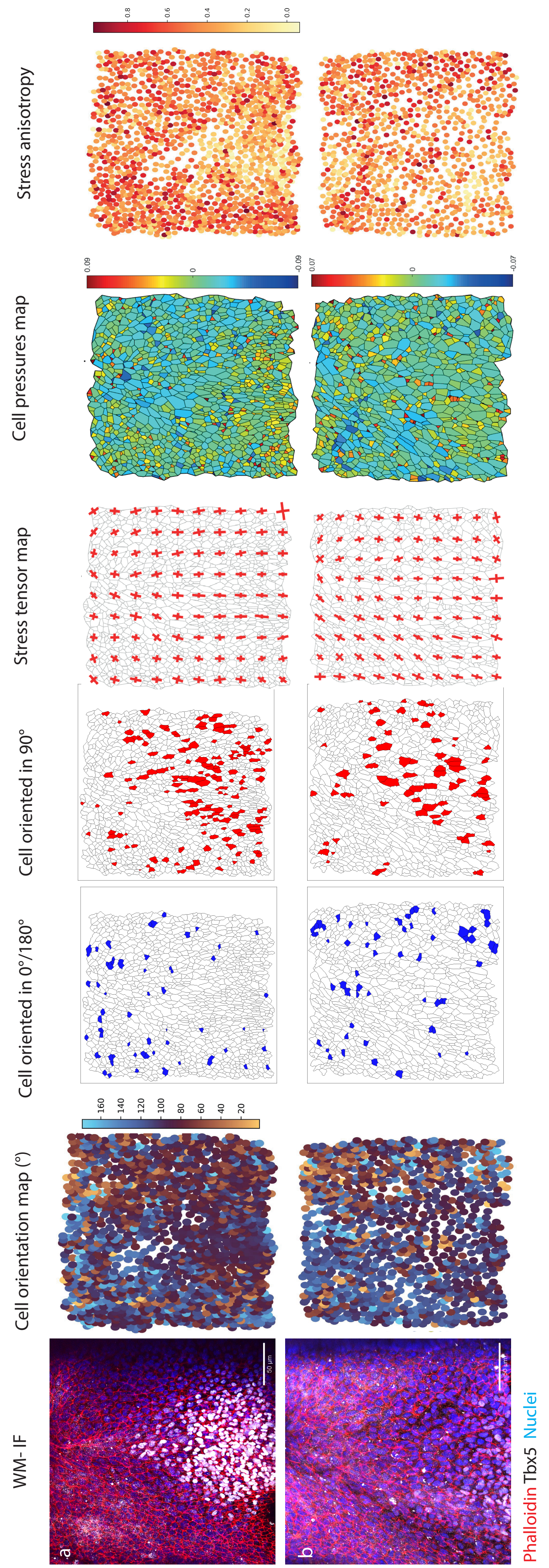

WM- IF
Cell orientation map (°)
Cell oriented in 0°/180°
Cell oriented in 90°
Stress tensor map
Cell pressures map
Stress anisotropy
a
b
Phalloidin Tbx5 Nuclei
50 µm

Fig. S11: Morphometrics reproducibility in E9.5 *Mef2cAHFCre;Tbx5$^{fl/+}$* embryos. a-b. Showing, for each embryo, in the following order: the apical projection of the dorsal pericardial wall after whole-mount immunofluorescence (phalloidin in red, nuclei in blue, TBX5 in white), the cell orientation map, cells oriented horizontally (in blue) along the left-right axis of the embryo on the segmentation channel, cells oriented vertically (in red) along the anterior-posterior axis of the embryo, the stress tensor map, the cell pressure map and the stress anisotropy map. N = 3.

Figure S12

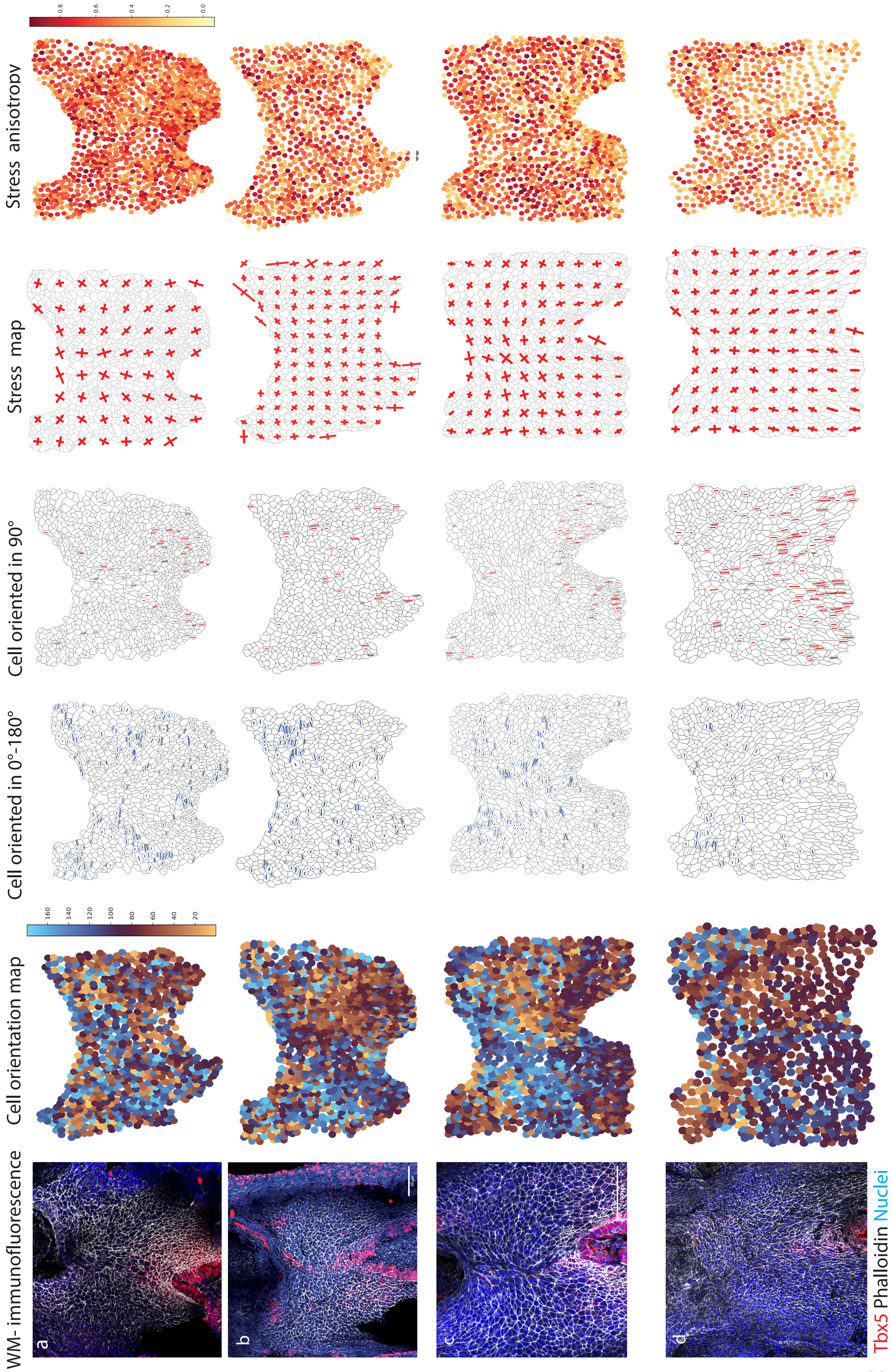
Stress anisotropy
Stress map
Cell oriented in 90°
Cell oriented in 0°-180°
Cell orientation map
WM- immunofluorescence
a
b
c
d
Tbx5 Phalloidin Nuclei
0.8
0.6
0.4
0.2
0.0
160
140
120
100
80
60
40
20

Fig. S12: Morphometrics of the dorsal pericardial wall of E9.5 embryos treated with the retinoic acid antagonist BMS493 in embryo culture. a-d. Showing, for each embryo, in the following order: the apical projection of the dorsal pericardial wall after whole-mount immunofluorescence (TBX5 in red, Golgi in green, phalloidin in white, nuclei in blue), the cell orientation map, cells oriented horizontally (in blue) along the left-right axis of the embryo on the segmentation channel, cells oriented vertically (in red) along the anterior-posterior axis of the embryo, the stress tensor map and the stress anisotropy map. N = 4 BMS493 treated embryos.

Figure S13

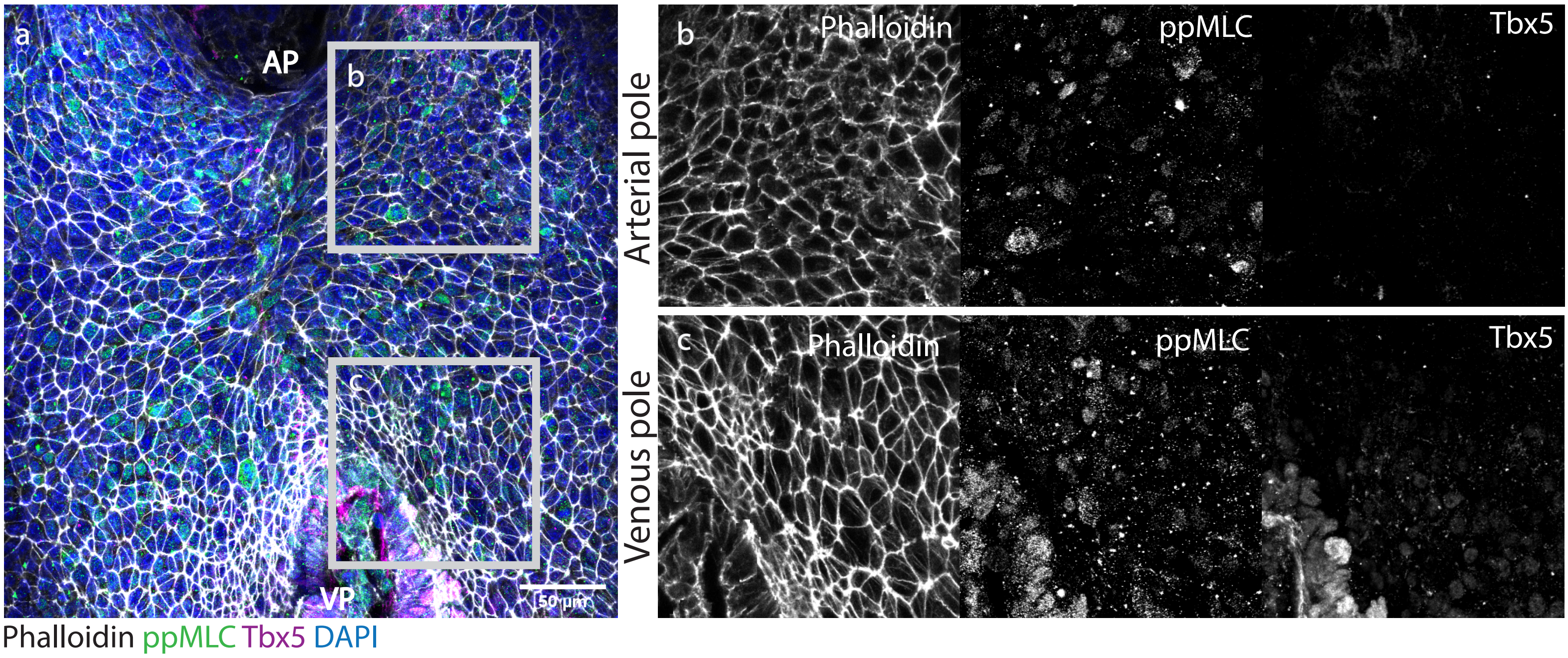

Fig. S13: Diphosphorylated Myosin light chain 2 (ppMLC) distribution in E9.5 embryos treated with retinoic acid inhibitor BMS493. a. Maximum intensity projection of the apical part of the epithelia **b.** Magnification of the aDPW region (box b in panel a). **c.** Magnification of the pDPW region (box c in panel a). Phalloidin (white), ppMLC (green) and TBX5 (red). N = 5 wild-type embryos; N = 4 BMS493 treated embryos.

Figure S14

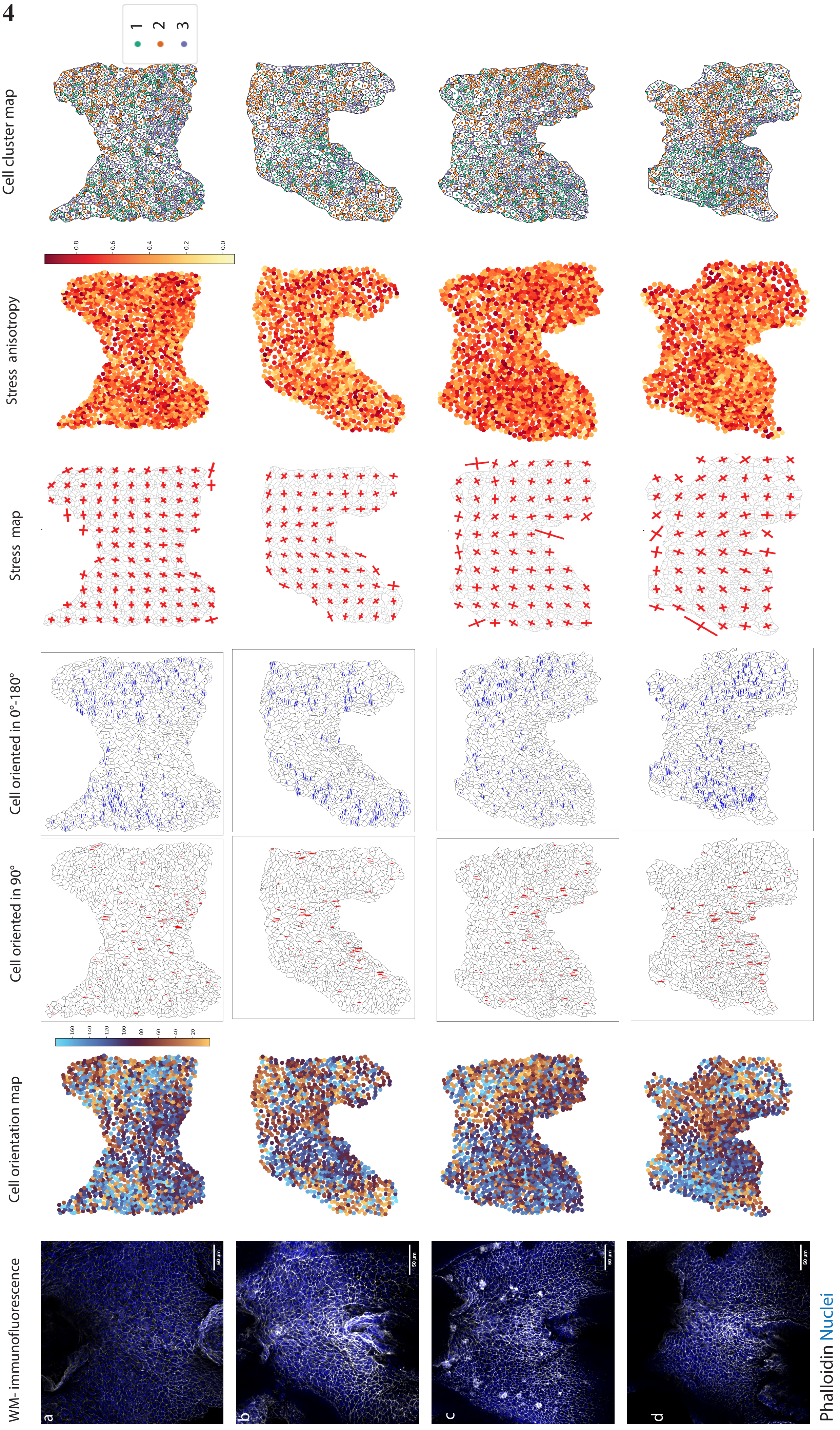
Cell cluster map
1
2
3
Stress anisotropy
0.8
0.6
0.4
0.2
0.0
Stress map
Cell oriented in 0°-180°
Cell oriented in 90°
Cell orientation map
160
140
120
100
80
60
40
20
WM- immunofluorescence
a
b
c
d
50 µm
Phalloidin Nuclei

Fig. S14: Morphometrics reproducibility in E9.5 *Nkx2.5*$^{-/-}$ embryos. a-d. Showing, for each embryo, in the following order: the apical projection of the dorsal pericardial wall after whole-mount immunofluorescence (phalloidin in white, nuclei in blue), the cell orientation map, cells oriented horizontally (in blue) along the left-right axis of the embryo on the segmentation channel, cells oriented vertically (in red) along the anterior-posterior axis of the embryo, the stress tensor map, the cell pressure map and the stress anisotropy map. N = 4 embryos, n = 5568 cells.

Figure S15

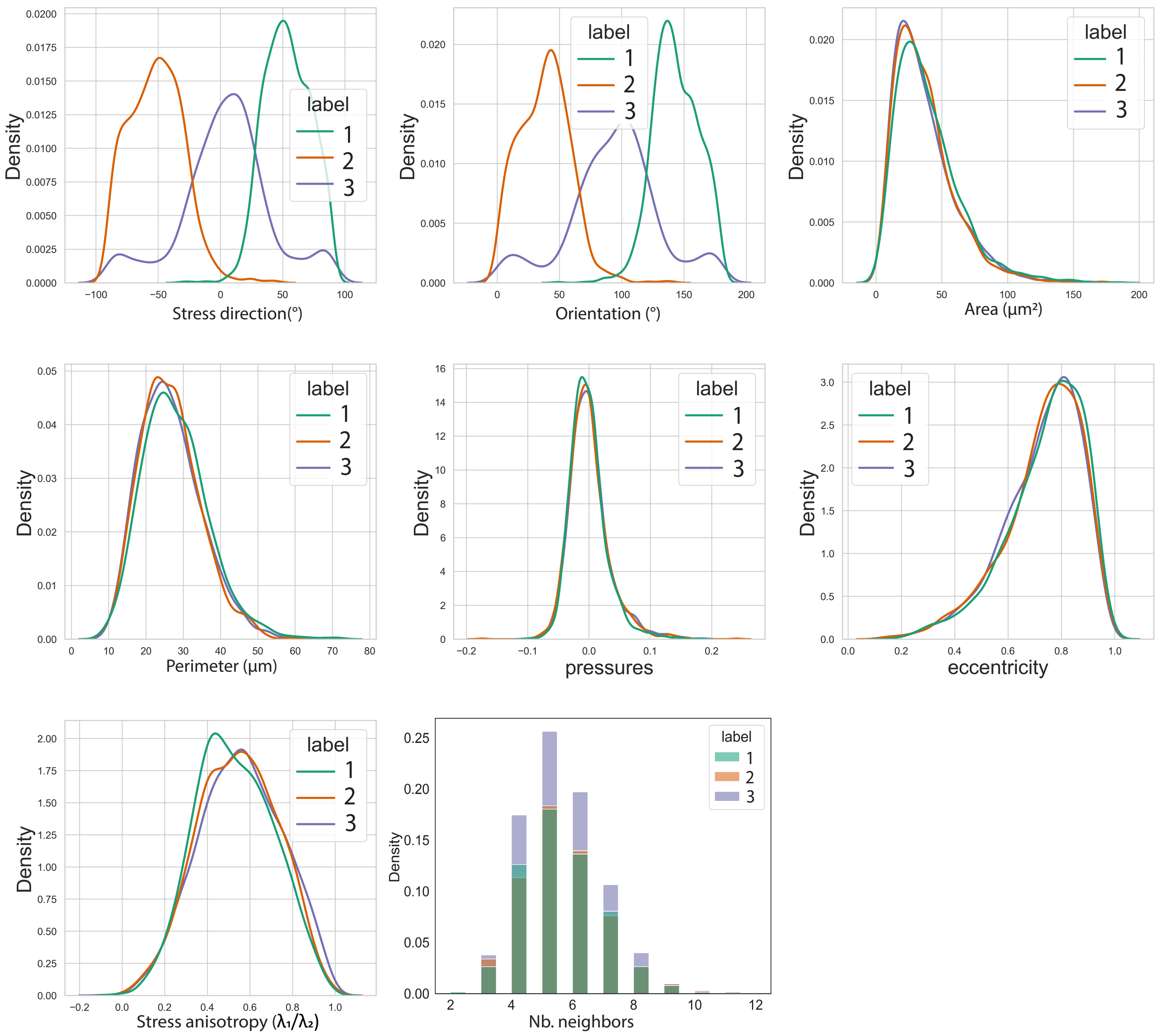

Fig. S15: Cluster signatures of *Nkx2.5*$^{-/-}$ embryos. Kernel density and histogram distributions of each morphometrical feature for *Nkx2.5*$^{-/-}$ embryos. Source data are provided as a Source Data file. Cluster 1 in green, cluster 2 in orange, cluster 3 in purple. N = 4 embryos, n = 5568 cells.

Figure S16

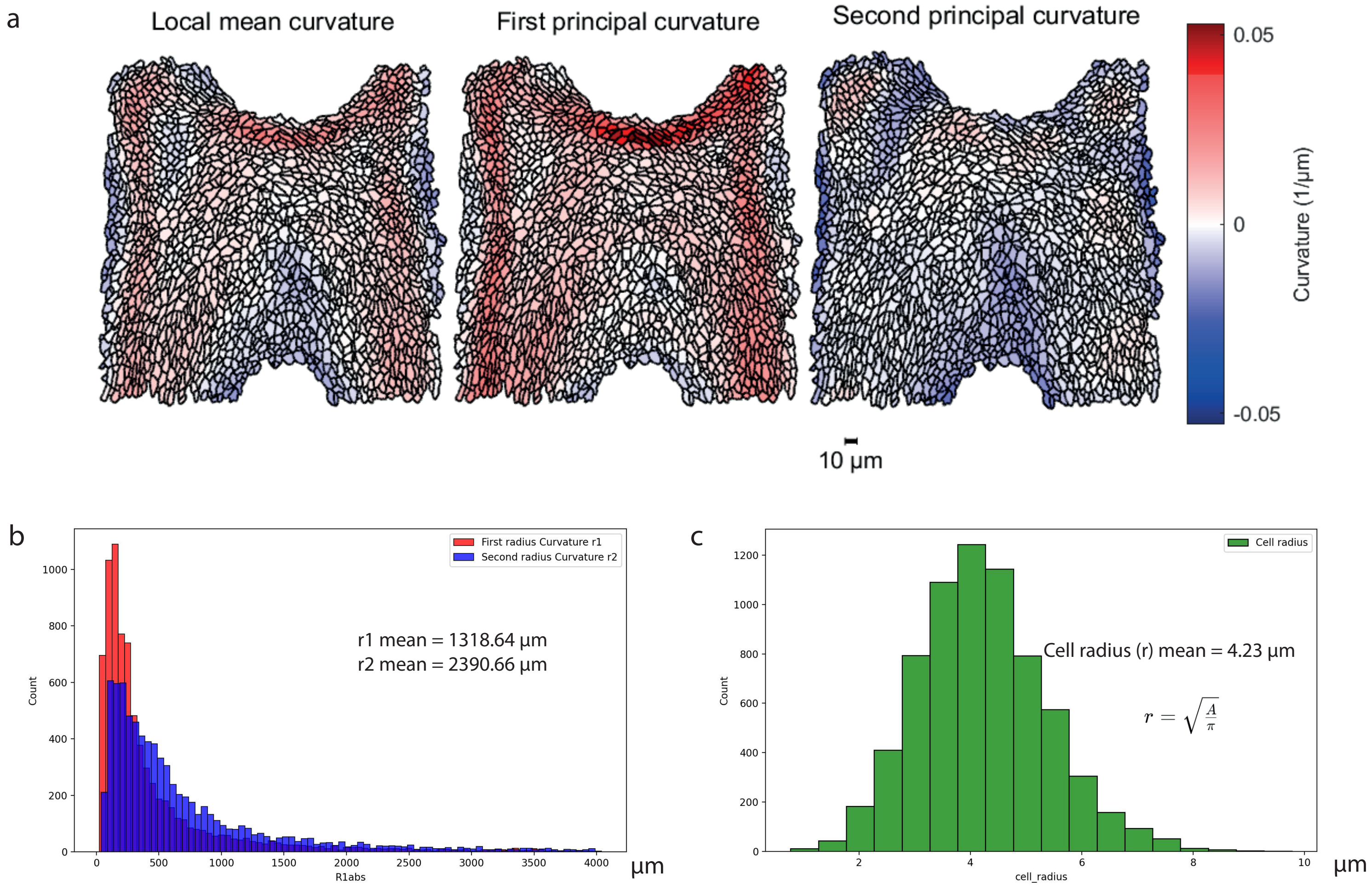

Fig. S16: Force inference curvature tests a. Map of the mean, first and second principal curvature of an E9.5 wild-type embryo. **b.** Histogram plot distribution of the absolute values for the first and second radius of curvature from all E9.5 wild-type embryos, with a threshold set at 4000 µm used for visualization purposes. **c.** Histogram plot showing the distribution of the cell radius from all E9.5 wild-type embryos. N = 6 embryos, n = 8618 cells.

Figure S17

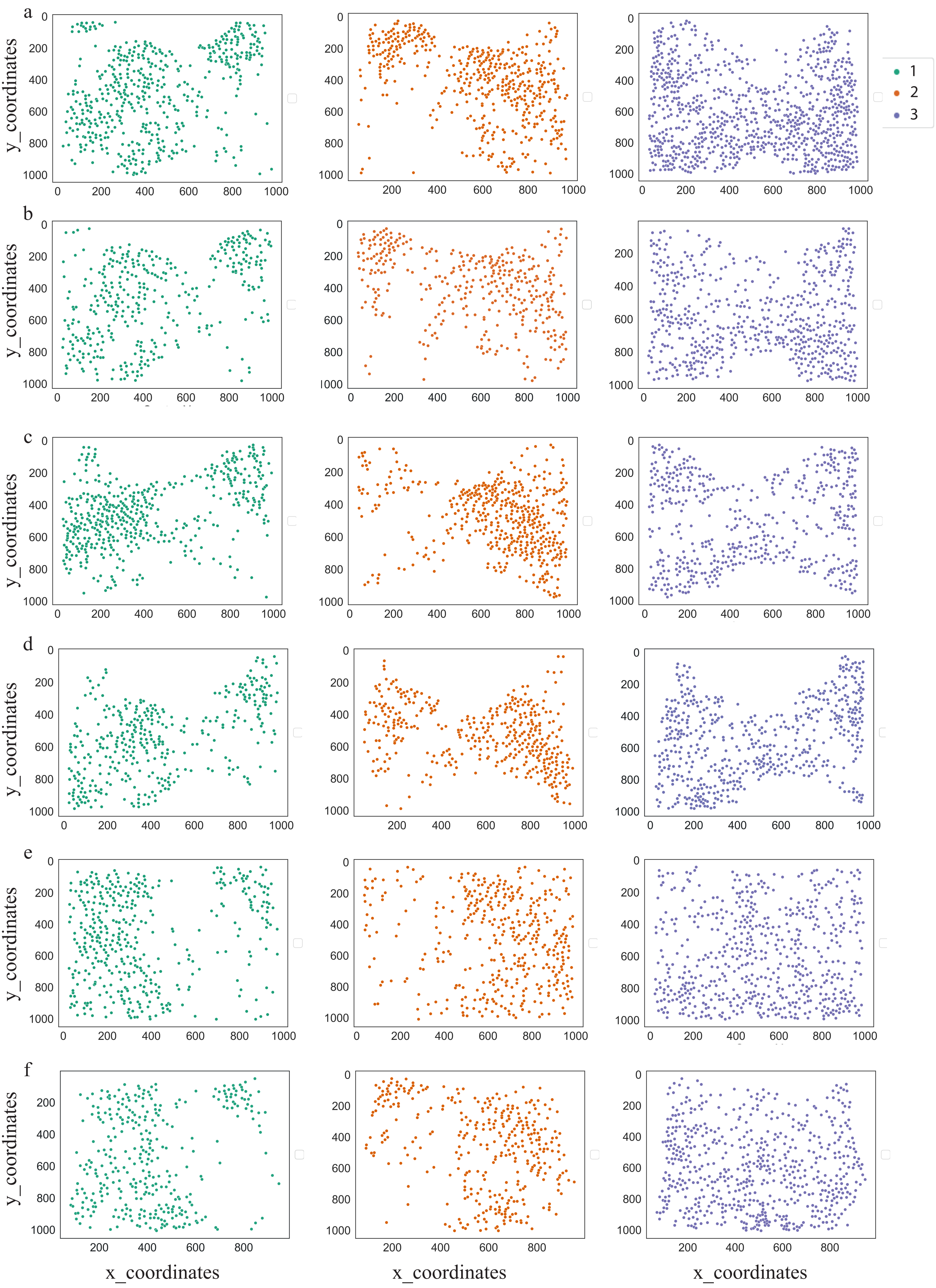

Fig. S17: Cluster maps for E9.5 wild-type embryos showing each cluster independently. a-f. Scatter plots with axes representing the x-y coordinates of the cells, showing each cluster independently**.** Cluster 1 in green, cluster 2 in orange, cluster 3 in purple. N = 6 embryos, n = 8618 cells.

Figure S18

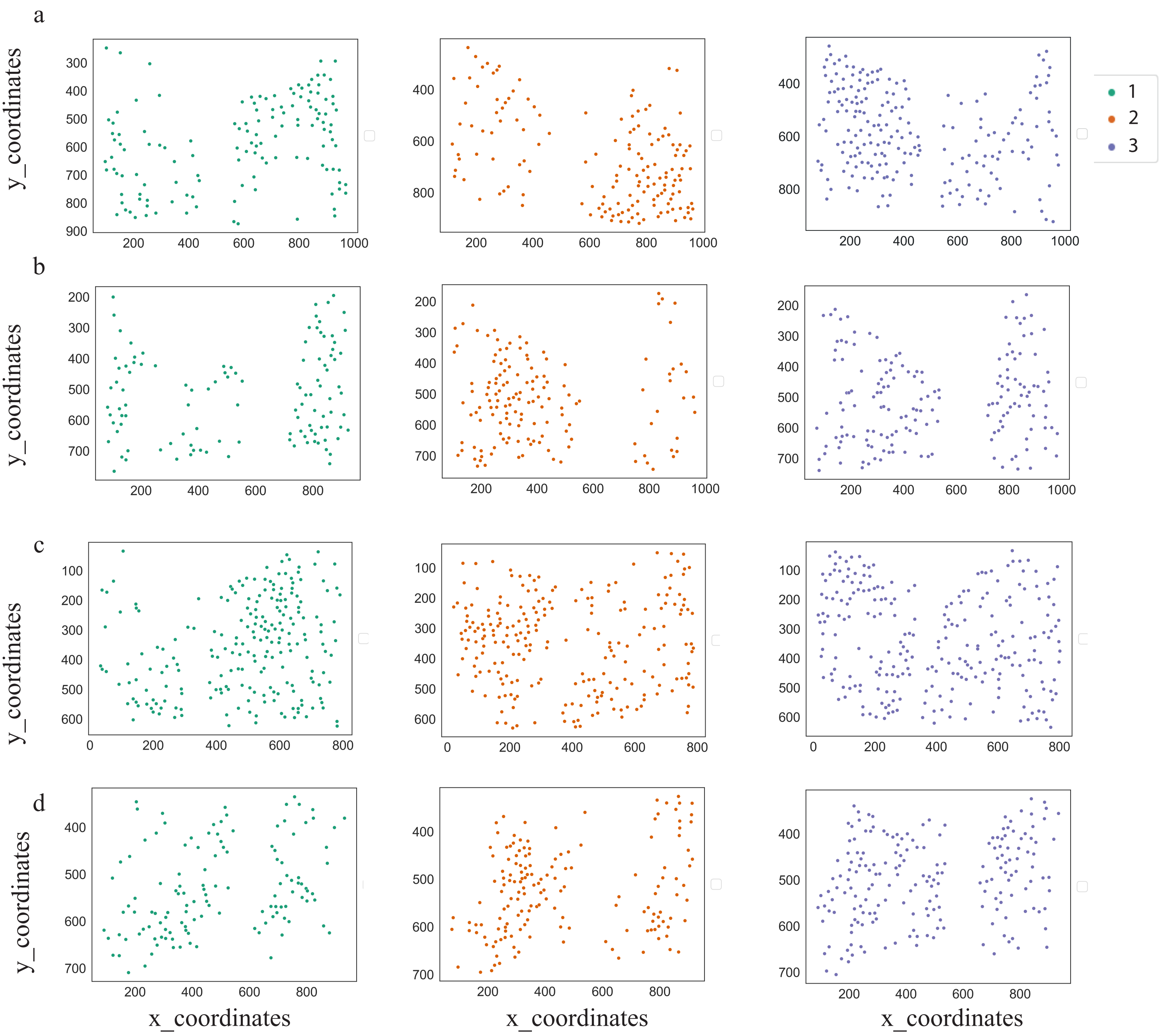

Fig. S18: Cluster maps for E8.5 wild-type embryos showing each cluster independently. a-d. Scatter plots with axes representing the x-y coordinates of the cells, showing each cluster independently. Cluster 1 in green, cluster 2 in orange, cluster 3 in purple. N = 6 embryos, n = 2994 cells.

Figure S19

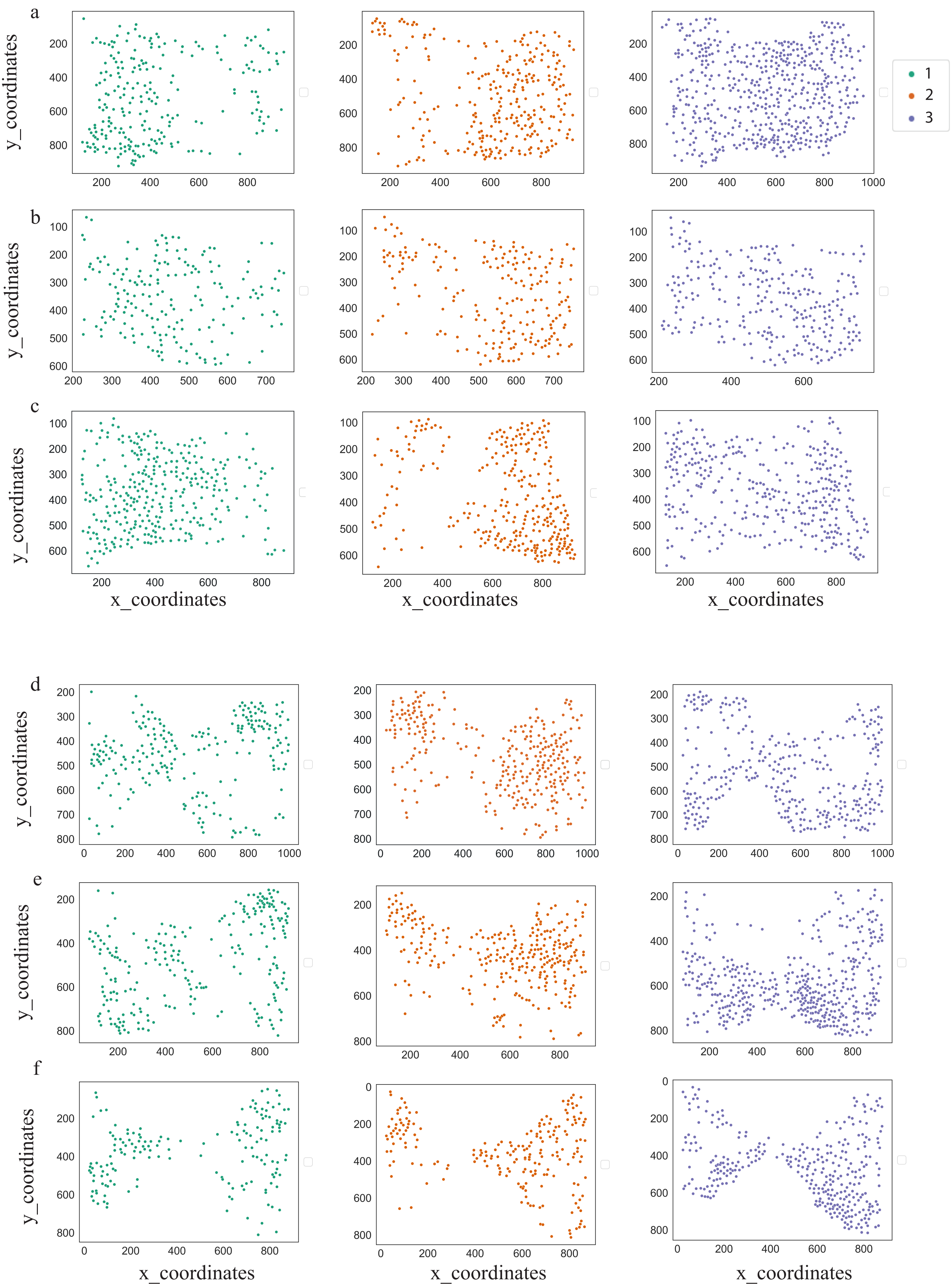

Fig. S19: Cluster maps for E9.5 *Mef2cAHFCre;Tbx5$^{fl/fl}$* and *Tbx1$^{-/-}$* embryos showing each cluster independently. a-c. Scatter plots with axes representing the x-y coordinates of the cells, showing each cluster independently for *Mef2cAHFCre;Tbx5$^{fl/fl}$* embryos and **d-f.** for *Tbx1$^{-/-}$* embryos. Cluster 1 in green, cluster 2 in orange, cluster 3 in purple. *Tbx1$^{-/-}$* : N = 3 embryos, n = 2551; *Mef2cAHFCre;Tbx5$^{fl/fl}$* : N = 4 embryos, n = 2809 cells.

Figure S20

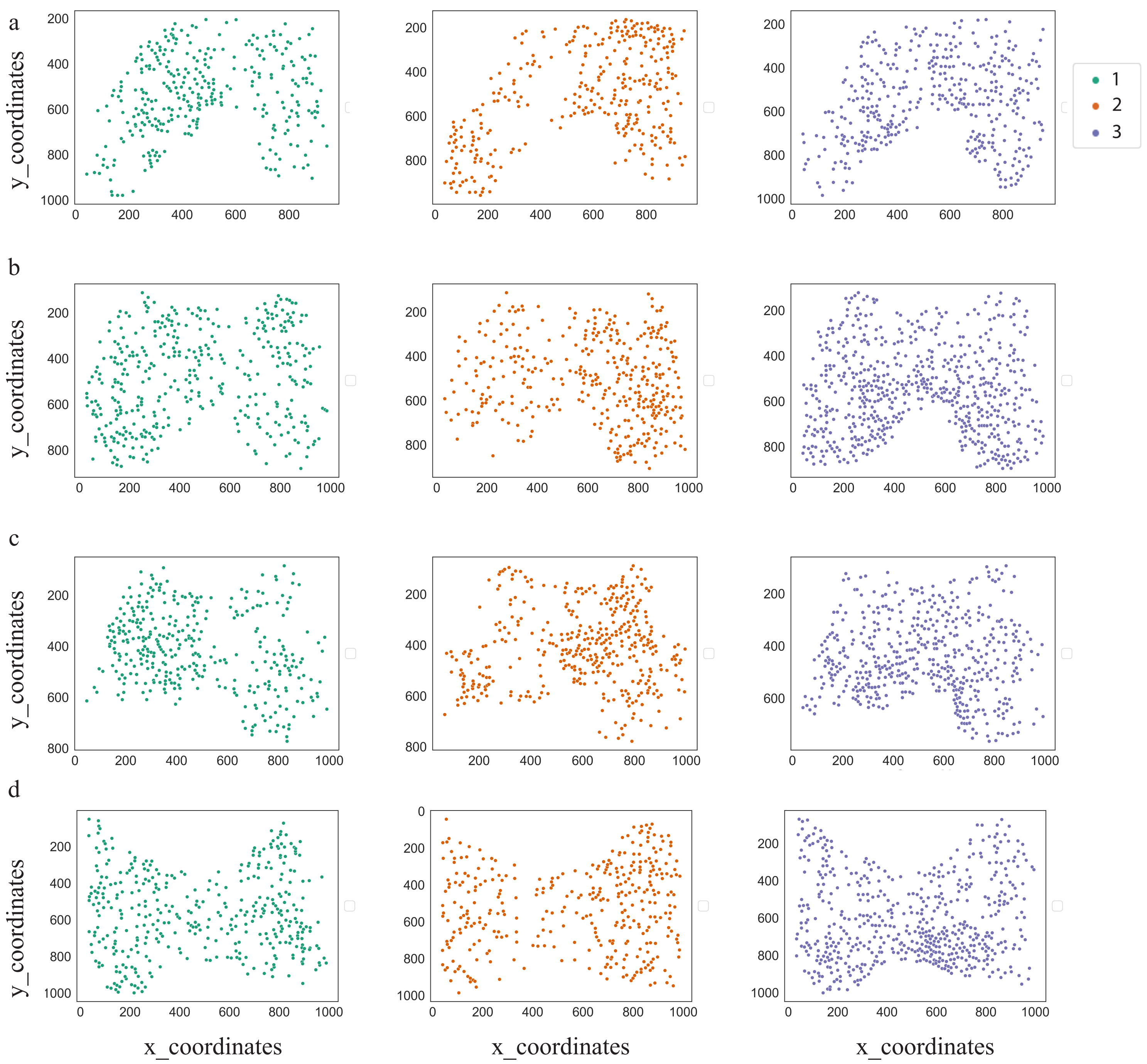

Fig. S20: Cluster maps for E9.5 *Nkx2.5*$^{-/-}$ embryos showing each cluster independently. a-d. Scatter plots with axes representing the x-y coordinates of the cells, showing each cluster independently**.** Cluster 1 in green, cluster 2 in orange, cluster 3 in purple. N = 4 embryos, n = 5568 cells.

Supplementary Table 1

Mouse line	Primer sense	Primer sequence	Product size
MefC-AHF-Cre	**FW**	5’ AGG TTC GTT CAC TCA TGG A 3’	Cre = 200 bp
	**RV**	5' TCG ACC AGT TTA GTT ACC C 3'	
Tbx5 $^{fl/fl}$	**FW**	5’ AGT CCA ATG ACT TAT CCT GGT GCG C 3’	WT = 203 bp; MUT = 232bp
	**RV**	5’ AAA TTC CAA CCC CTT CCA CAG ATC GG 3’	
Tbx1 $^{+/-}$	**a**	5’ CCC AAG GTG GTC TAG CTC TC 3’	WT = 241 bp; MUT = 300 bp
	**b**	5’ CCT TCC TCT GTA GCC ATA CTG C 3’	
	**c**	5’ ATC TCT CGG GGG ATC ATT C3’	
Nkx2-5 $^{-/-}$	**RW**	5' GAACCTGGAGCAGCAGCAGCGTAGC 3'	WT = 308 bp; MUT = 1376bp
	**FW**	5' CAGAAGGGAAGAGCTTGAGGTTCTC 3'	

Supplementary Table 1: List of PCR primers used for genotyping
